# Human Herpes Virus—Six Related Clinical and Functional Implications in Lung Transplant Patients: Bronco Alveolar Lavage Analysis, Coinfections, Rejection, and Survival

**DOI:** 10.3390/pathogens14111157

**Published:** 2025-11-13

**Authors:** Paolo Solidoro, Antonio Curtoni, Costanza Perotti, Camilla Perotti, Nour Shbaklo, Francesca Sidoti, Mauro Mangiapia, Francesco Giuseppe De Rosa, Silvia Corcione, Massimo Boffini, Matteo Marro, Cristina Costa, Rocco Francesco Rinaldo

**Affiliations:** 1 Division of Respiratory Medicine, Cardiovascular and Thoracic Department, AOU Città Della Salute e Della Scienza di Torino, University of Turin, 10126 Torino, Italymmangiapia@cittadellasalute.to.it (M.M.); roccofrancesco.rinaldo@unito.it (R.F.R.); 2 Medical Sciences Department, University of Turin, 10126 Torino, Italy; 3 Division of Virology, Department of Laboratory Medicine Public Health and Pediatrics, AOU Città Della Salute e Della Scienza di Torino, University of Turin, 10126 Torino, Italy; antonio.curtoni@unito.it (A.C.); cristina.costa@unito.it (C.C.); 4 Department of Public Health and Paediatrics, University of Turin, 10124 Torino, Italy; 5 Department of Neurosciences, Rita Levi Montalcini, University of Turin, 10126 Torino, Italy; camilla.perotti@unito.it; 6 Unit of Infectious Diseases, Department of Medical Sciences, University of Turin, 10149 Torino, Italy; 7 Cardiac Surgery Division, Cardiovascular and Thoracic Department, AOU Città Della Salute e Della Scienza di Torino, 10124 Torino, Italymatteo.marro1@gmail.com (M.M.); 8 Surgical Sciences Department, AOU Città della Salute e della Scienza di Torino, University of Turin, 10126 Torino, Italy

**Keywords:** human herpes virus 6, lung transplantation, infection, epidemiology, BAL

## Abstract

Human herpesvirus 6 (HHV-6) is a common virus that can reactivate in immunocompromised patients, including lung transplant (LT) recipients. This study aimed to evaluate the clinical and functional implications of HHV-6 infection in LT patients through a retrospective analysis of 175 individuals who underwent lung transplantation at the City of Health and Sciences of Turin between 2014 and 2023. Surveillance bronchoscopies—including bronchoalveolar lavage (BAL) and transbronchial biopsies—were performed at scheduled intervals over a two-year period to detect HHV-6 and other pathogens, and to assess acute rejection. Spirometries were performed to evaluate graft function. Among the cohort, 33% of 822 BAL samples tested were positive for HHV-6, with a notable association between high viral load (≥500 copies/mL) and the development of post-transplant lymphoproliferative disorder (PTLD) (13% vs. 1%, *p* = 0.02) at 1 month and (9% vs. 1%, *p* = 0.026) at 12 months. Co-infection with CMV (78% in positives vs. 55% in negatives; *p* = 0.006), Epstein–Barr virus (EBV) (35% vs. 16%; *p* = 0.010), and bacterial and fungal infection (specifically, a higher rate of isolation of *Achromobacter xylosoxidans* (13%), *p* = 0.010) was frequently observed in conjunction with HHV-6 positivity. Notably, patients with at least one HHV-6 positive BAL exhibited a significant reduction in forced vital capacity (FVC) at multiple follow-up points, FVC 82% in positives vs. 92% in negatives (*p* = 0.038) at 4 months and 87% vs. 98% *p* = 0.033 at 8 months and 87% vs. 99% *p* = 0.038 at 24 months. No direct associations with acute rejection or overall survival were found. By means of this study, we provide a wide overview of HHV-6 in lung transplant recipients, filling in a gap of evidence in the field. We report a remarkable incidence and a significant association with acknowledged clinically relevant viral infections, PTLD, and functional tests decline, with no association with mortality.

## 1. Introduction

Lung transplantation (LT) represents the ultimate therapeutic option for patients with advanced, end-stage respiratory diseases [1]. The risk of infection in LT recipients is higher than in other solid organ transplant settings [2,3] and monitoring herpesvirus infections plays a pivotal role in the management of these patients [4].

Human herpesvirus 6 (HHV-6) is globally distributed, with an estimated adult seroprevalence exceeding 95% [5,6]. Like other herpesviruses, HHV-6 establishes lifelong latency, with the potential for recurrent reactivations [7,8]. Demonstrating a direct causal role of HHV-6 in many diseases remains challenging due to its ubiquitous nature and the chronic course of the infection [9,10].

Primary HHV-6 infection, while a well-recognized cause of exanthema subitum in young children, is uncommon in adults [9]. However, reactivation can be triggered by immunosuppression [11], occurring frequently after transplantation and potentially leading to encephalitis, cytopenia, hepatitis, and pneumonitis—particularly in the setting of hematopoietic stem cell transplantation (HSCT) [9,12,13,14,15,16].

While HHV-6 detection after HSCT has been linked to non-relapse mortality and overall mortality [17], data regarding HHV-6 infection and its clinical implications following LT remain scarce. Given that airway infections constitute one of the major challenges in the management of transplant recipients [16], bronchoscopic surveillance represents a valuable strategy for the detection and longitudinal monitoring of HHV-6 and other opportunistic pathogens. Nevertheless, high-quality studies directly comparing outcomes between surveillance bronchoscopy and clinically indicated procedures for diagnosing rejection and infection are still lacking [18].

## 2. Materials and Methods

A retrospective study was conducted that included patients who underwent lung transplantation at the City of Health and Sciences of Turin between 2014 and 2023. Data on bronchoalveolar lavage (BAL) surveillance in the first 24 months following transplantation were collected.

A bronchoalveolar lavage (BAL) sample was considered positive for viral infection (including HHV-6 and other detected viruses) if viral DNA was identified by polymerase chain reaction (PCR). The detection of viral DNA in the BAL fluid was interpreted as indicative of the presence of the virus.

The transplant center’s surveillance protocol for the early detection of rejection and infectious complications includes scheduled transbronchial biopsies and BAL at months 1, 4, 8, 12, 18, and 24 post-transplant [19].

BAL samples were analyzed at the microbiology laboratory of the center. Flexible video bronchoscopes were used, and three 50 mL aliquots of saline solution were instilled and aspirated from subsegmental bronchi in the target areas (middle lobe or lingula) [20].

BAL samples were analyzed at the microbiology laboratory of the center. For each BAL sample, the following microbiological data were considered and analyzed: studied bacterial, fungi growth on appropriate media, and herpesvirus amplification results by quantitative real-time polymerase chain reaction. In particular, HHV-6, EBV, CMV, HSV-1, and HSV-2 were detected by HHV6 ELITe MGB Kit, EBV ELITe MGB Kit, CMV ELITe MGB Kit, HSV1 ELITe MGB Kit, and HSV2 ELITe MGB Kit (ELITechGroup, Torino, Italy), respectively, on 7500 Fast Dx system (ThermoFisher, Waltham, MA, USA).

Additionally, respiratory function parameters were recorded—specifically, forced expiratory volume in one second (FEV1) and forced vital capacity (FVC). The occurrence of acute rejection was assessed through transbronchial biopsies (TBB), along with the incidence of neoplasms and mortality at two years post-transplant.

The population was subsequently stratified into two subgroups based on the viral load of HHV-6 detected in the BAL: one group consisting of patients with a viral load >500 copies/mL and another group with a viral load < 500 copies/mL or negative.

## 3. Statistical Analysis

The population was stratified into two subgroups based on the viral load of HHV-6 detected in the BAL: one group consisting of patients with a viral load ≥ 500 copies/mL (HHV-6 positive patients) and another group with a viral load < 500 copies/mL or negative (HHV-6 negative patients). Data were analyzed using SPSS version 29 to compare the results between HHV6 positive and negative patients. A significance threshold of *p* < 0.05 was used to determine statistically significant differences. Microbiological results, respiratory function parameters, and information regarding rejection, neoplasms, and mortality were reported and compared between the two subgroups to assess the impact of HHV-6 viral infections on clinical outcomes and mortality post-transplant. Comparisons were made using the χ^2^ test for categorical variables and the Kruskal–Wallis test or Student’s *t*-test, depending on the distribution, for continuous variables. Survival analysis was conducted using the Kaplan–Meier method to estimate survival curves. Survival time was defined as the interval between transplantation and death.

## 4. Results

**Figure 1:**
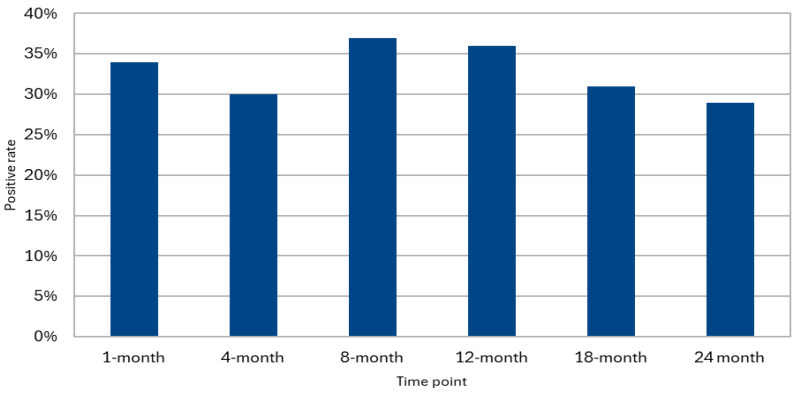
HHV-6 positivity in surveillance BAL.

In total, 217 patients undergoing lung transplantation were included. Of them, 175 (81%) underwent at least one BAL surveillance in the first 24 months following the transplant. Among these, 131 patients (75%) had at least one BAL sample positive for HHV-6 during the follow-up period (Figure 1). In our cohort, the distribution of BALs performed at the various time points outlined in the surveillance protocol were as follows: 146 (83%) patients underwent BAL at 1 month post-transplant, 159 (91%) at 4 months, 147 (84%) patients at 8 months, 140 (80%) patients at 12 months, 121 (69%) patients at 18 months, and 109 (62%) patients at 24 months (Table 1).

In a cohort of 175 patients who underwent at least one BAL, the mean age was 51 years, with a majority being male N = 108 (62%), as seen in Table 1. The primary indications for lung transplantation included interstitial lung diseases N = 77 (44%), chronic obstructive pulmonary disease N = 45 (26), and cystic fibrosis/other bronchiectasis N = 38 (22%). Most patients received bilateral transplants N = 144 (82%). In total, 18 (10%) patients had a solid post-transplantation neoplasm. Acute rejection episodes were most prevalent at 1 month post-transplant N = 49 (35%), with varying degrees of severity reported at subsequent time points. At the 24-month follow-up, the mean FEV1 and FVC percentages predicted were 79 ± 23 and 87 ± 23, respectively, while the mortality rate at 24 months was N = 34 (19%).

**Figure 2:**
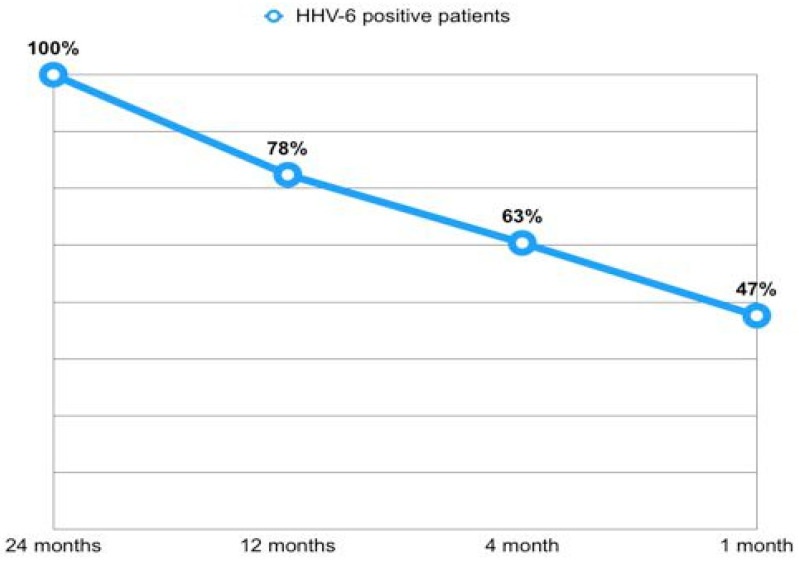
Early isolation rates of HHV-6 in patients positive at 24 months.

Overall, out of 822 available BAL samples, 33% tested positive for HHV-6 (N = 270). Among the 131 patients who were positive for HHV-6 in at least one BAL, 105 (80.2%) underwent at least two surveillance BALs during the observation period. Of these, 40 patients (38.1%) tested positive for HHV-6 in at least 50% of the BALs. Additionally, among the patients with HHV-6 isolation in the BAL at 1 month post-transplant, 44 continued bronchoscopy surveillance until at least the eighth month. Of these, 23 patients (52%) maintained positivity for HHV-6 in both the first and the last available BAL. Among the 32 patients who completed the 24 months follow-up and tested positive for HHV-6 at that time point, 15 individuals (47%) had already shown positivity in a previous sample within the 1-month BAL, with an increasing rate of positivity through time (Figure 2).

## 5. Comparison Between Subgroups

At the 1-month surveillance BAL, dividing our population into subjects with HHV-6 isolation in BAL (Table 2) versus patients with negative BAL for HHV-6, there was a statistically significant association with EBV isolation (55% vs. 20%, *p* < 0.001), and the presence of bacterial and fungal co-infection (10% vs. 1%, *p* = 0.008). We did not find a significant difference in terms of bacterial isolation in general, but, specifically, a higher rate of isolation of *Achromobacter xylosoxidans* was noted in HHV-6 positive patients compared to HHV-6 negative patients (13% vs. 2%, *p* = 0.010) (Table 2).

No significant associations emerged regarding acute rejection, FVC values, FEV1, or 24-month survival (Table 2). Regarding HHV-6 isolation in the surveillance BAL at 4 months, a statistically significant association was found between HHV-6 positivity and the development of PTLD (*p* = 0.028). Furthermore, the presence of HHV-6 at 4 months significantly correlated with the co-detection of CMV (78% in positives vs. 55% in negatives; *p* = 0.006) and EBV (35% vs. 16%; *p* = 0.010). No significant associations were found with the occurrence of acute rejection, respiratory function changes (FEV1 and FVC values), or 2-year survival post-transplant (Table 2).

Among subjects with HHV-6 positivity in BAL at 8 months post-transplant and at 12 months, a statistically significant association emerged with the concomitant isolation of EBV (44.4% vs. 24%, *p* = 0.009 at 8 months; 46% vs. 22%, *p* = 0.003 at 12 months). No significant associations were found with other variables.

Regarding HHV-6 positive subjects at the BAL at 18 months compared to negative subjects, no significant associations emerged with any of the above variables, including co-infection with CMV and EBV.

Analysis of viral persistence showed that 78.1% of those positive at month 24 had at least one positive sample within the first 12 months (Figure 2). On the other hand, among patients with 1-month HHV-6 positivity, 52.2% remained positive in their last available BAL sample.

Finally, among subjects with HHV-6 present in BAL at 24 months post-transplant compared to patients with negative BAL, a statistically significant association was found with the concomitant isolation of EBV (53% vs. 27%, *p* = 0.010) and HSV1 (22% vs. 4%, *p* = 0.003). No significant associations were found with other variables.

In detail, stratifying our population between patients with more than 500 copies/mL of HHV-6 DNA in BAL versus patients with negative BAL for HHV-6 or with less than 500 copies/mL, a significant association was highlighted at 1 month between the presence of high-load HHV-6 and the future onset of PTLD (13% vs. 1%, *p* = 0.002). At 4 months, a statistically significant association was noted between a high load of HHV-6 and co-infection with *Achromobacter xylosoxidans* (12% vs. 1%, *p* = 0.010).

Additionally, at 12 months, a statistically significant association was again observed between an HHV-6 load in BAL greater than 500 copies/mL and the development of PTLD (9% vs. 1%, *p* = 0.026). Additional statistically significant associations emerged in the analysis of BAL performed at 24 months post-transplant, where a viral load of HHV-6 greater than 500 copies/mL was correlated with the concomitant presence of EBV (71% vs. 32%, *p* = 0.036) and HSV-2 (14% vs. 1%, *p* = 0.011).

## 6. HHV-6 and Respiratory Function

**Figure 3:**
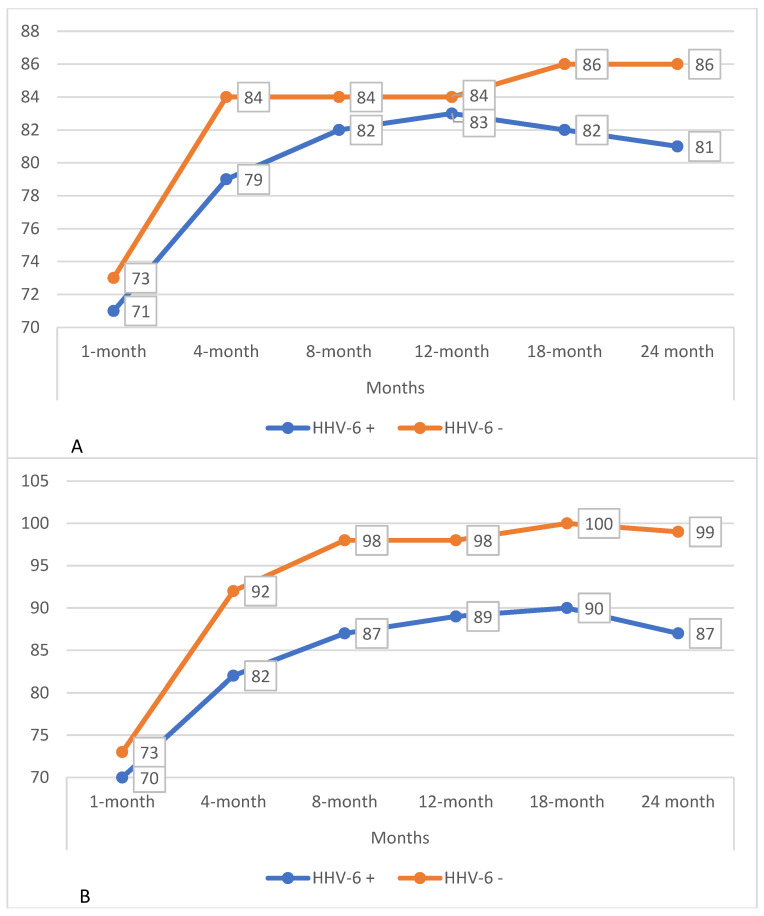
(**A**) Trends in FEV1% predicted in patients with and without HHV-6 isolation in BAL during the first 24 months post-transplant. (**B**) Trends in FVC % predicted in patients with and without HHV-6 isolation in BAL during the first 24 months post-transplant.

Within the cohort, 97 patients who completed surveillance bronchoscopy from 1 month post-transplant to 24 months post-transplant were selected. These patients were divided based on HHV-6 positivity in at least one BAL, regardless of load, during the two years of follow-up. Within the subgroups, the trend of respiratory functionality was assessed in terms of FEV1 and FVC percentage predicted (Table 3, Figure 3).

**Figure 4:**
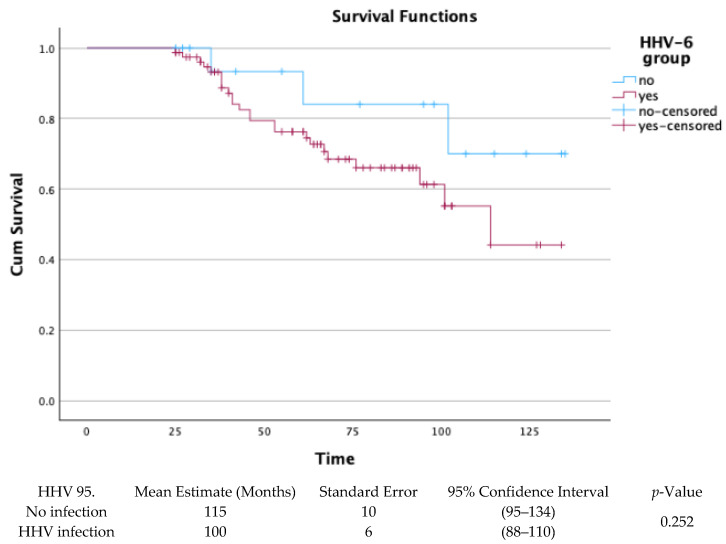
Kaplan–Meier for HHV infection or no infection in BAL-surveilled patients.

The long-term survival of the 97 patients who completed surveillance bronchoscopy from 1 to 24 months post-transplant, was assessed through Kaplan–Meier survival analysis. There was no statistical significance between groups with HHV infection or no infection (*p*-value = 0.252), as shown in Figure 4.

## 7. Discussion

In this study, which was conducted on a cohort of LT recipients undergoing surveillance bronchoscopies during the first 24 months post-transplant, the following three main findings emerged:

1. The frequent association between HHV-6 detection and EBV co-infection in BAL samples, observed at multiple follow-up time points;
2. The association between high HHV-6 viral load (>500 copies/mL) and the development of PTLD, particularly in BAL samples collected at months I and XII post-transplant;
3. A significant reduction in FVC values among patients with at least one HHV-6-positive BAL sample at multiple follow-up time points.

HHV-6 reactivation is a common occurrence in immunocompromised patients; however, its specific role in LT recipients and its potential prognostic implications remain largely unclear.

Clinically, screening for HHV-6 infection is not recommended by guidelines and most patients that are HHV-6 positive are reported in the literature as asymptomatic. Nevertheless, in the suspicion of clinically relevant HHV-6 infection, quantitative PCR in blood is the preferred method for diagnosis, although without a clear agreement on the cut-offs [22].

Scheduled BAL procedure protocol is a unique opportunity to assess the presence of viruses, particularly herpesviruses, in the alveolar microenvironment. Our study mainly aimed at exploring the role of HHV-6 presence, detected via BAL, and its relation to immunological and infectious outcomes in LT recipients.

In fact, possessing a role as a driver agent of immunological responses related to rejection has been hypothesized for one of the most clinically relevant herpesviruses, CMV, even in the absence of clinically relevant infections [23,24], prompting debate on the overall necessity of aggressive prophylactic strategies [25].

On the other hand, viral load, specifically *Torquetenovirus* load (TTV-load), a non-pathogenic, highly prevalent virus with a notable presence in the human virome [26], has been proposed as a marker of immune status in different kind of SOT (including lung), mostly measured via blood levels, but also via BAL [27,28,29]. However, Van Rijn et al. showed, in their metanalysis, how blood TTV-load measured within the first two years after SOT is associated with a higher risk of infection and a reduced risk of allograft rejection, though highlighting a substantial risk of bias in the studies evaluated [30].

In our study, 33% of the 822 BAL samples collected within the first 24 months post-LT tested positive for HHV-6. This prevalence is consistent with prior reports in solid organ transplantation, where the incidence of HHV-6 infection ranges from 10% to 60%, depending on patient population characteristics and laboratory methodologies [13].

For lung transplantation specifically, available data are limited and mostly derived from smaller cohorts. For instance, a previous study from our own center reported HHV-6 detection in 21.1% of BAL samples^4^, while other authors have described rates between 20% and 30% [31,32]. The largest body of evidence on HHV-6 derives from hematopoietic stem cell transplantation (HSCT) studies, where reactivation rates can reach 35–63% [33,34,35].

In our survival analysis, restricted to patients who completed surveillance bronchoscopies up to month 24, no statistically significant difference was observed between HHV-6-positive and HHV-6-negative patients (mean survival: 100 vs. 115 months; *p* = 0.252), supporting the minor direct role of HHV-6 positivity in mortality.

Regarding the viral persistence among patients with HHV-6 positivity, the high rates of patients featuring a positive BAL for the virus, may indicate that, at least for a subset of patients, HHV-6 detection is not a transient event but may rather suggest features of persistence.

A significant finding was the high frequency of viral co-infections, particularly between HHV-6 and EBV. This observation is consistent with previous evidence showing a strong association between these two herpesviruses in BAL samples from LT recipients [33,36,37]. These clinical data are supported by in vitro studies demonstrating that HHV-6 infection can induce EBV reactivation [38]. Such evidence supports the hypothesis of biological interactions among herpes viruses, which share the ability to establish latency and to reactivate under immunosuppressive conditions.

To further explore the pathogenetic role of HHV-6, we stratified patients by viral load, adopting a threshold of 500 copies/mL in BAL fluid, in line with a recent study identifying 578 copies/mL as the cut-off associated with detectable viral transcripts, and increased risk of overall mortality and death from respiratory failure [39]. In our cohort, this stratification revealed a significant association between high HHV-6 load and PTLD development in BAL samples from 1 month (*p* = 0.002) and 12 months (*p* = 0.026).

Given the well-established link between EBV and PTLD [30,40], it is plausible that HHV-6 could act as a cofactor in lymphoproliferative disease development. Indeed, as previously mentioned, HHV-6 can trigger EBV replication and may, therefore, contribute to the pathogenesis of EBV-associated disorders [38]. Nevertheless, when stratified by viral load, the HHV-6 and EBV association was confirmed only at 24 months, likely due to the small size of the analyzed subgroup.

Interestingly, no association emerged between HHV-6 detection and acute rejection, either at individual follow-up points or in aggregated analyses. Consistently, a previous study from our center [37] found no significant relationship between HHV-6 detection in transbronchial biopsies and acute rejection, despite a significant association with the histological features of interstitial pneumonia. In contrast, other studies have suggested a possible involvement of HHV-6 in the development of bronchiolitis obliterans [32], although these findings have not been replicated by others [31]. In the HSCT setting, by contrast, several studies have reported an association between HHV-6 infection and severe complications, such as GVHD [32,36,41].

Regarding lung function, patients with at least one HHV-6-positive BAL sample exhibited a significant reduction in percent-predicted FVC at months 4, 8, and 24 compared with persistently negative individuals, while no differences were observed in FEV1 values. These findings suggest the potential role of HHV-6 in impairing vital capacity, possibly mediated by chronic inflammation of the airways or lung parenchyma.

The limitations of the study include the retrospective and single-center design that may have limited the generalizability of the findings to broader populations. Additionally, the absence of correlative clinical or radiological data restricts the ability to comprehensively evaluate the clinical impact of HHV-6 infection per se on patient outcomes, although we hypothesize a more prevalent role as cofactor for development of different conditions.

The test we utilized would not allow us to distinguish between HHV-6 A and HHV-6 B. Although this distinction has clinically relevant implications when patients present the signs of a clinically relevant infection [33], we believe that this did not interfere with our exploratory aims and interpretation [42]. Surely, including this aspect in future studies is warranted.

In conclusion, our results indicate that HHV-6, frequently detected in BAL samples after lung transplantation, exhibits the feature of persistence and engages in viral interactions that may modulate clinical outcomes. The guidance on surveillance tests is still lacking and viremia monitoring is not recommended in SOT recipients’ management, nor in prophylaxis or in pre-emptive therapy [11]. Our exploratory study adds to the idea of assessing the viral load of the alveolar microenvironment as a marker of immunological status. Thus, while direct clinically relevant recommendations cannot be drawn from this data, further studies may be of use, to shed light on the role of BAL analysis in guiding immunosuppression-related clinical decisions.

## Figures and Tables

**Table 1:** Descriptive characteristics of patients who underwent at least one BAL.

Variable	Total N = 175 (100%)
Age	51 ± 14
Gender:	
Male	108 (62%)
Female	67 (38%)
Transplant indication:	
COPD	45 (26%)
Cystic fibrosis/other bronchiectases	38 (22%)
Interstitial lung diseases	77 (44%)
Cardiopathy	3 (2%)
A1AT deficiency	4 (2%)
Pulmonary hypertension	7 (4%)
Acute respiratory distress syndrome	1 (1%)
Pulmonary transplant:	
Monolateral	31 (18%)
Bilateral	144 (82%)
Other organ transplantation	6 (3%)
Post-transplantation neoplasm:	
Solid	18 (10%)
Post-Transplant Lymphoproliferative Disorder	3 (2%)
Acute TNN rejection	
1 month: (N = 141)	49 (35%)
A1	25 (51%)
A2	21 (43%)
A3	3 (6%)
A4	0
4 months: (N = 154)	38 (25%)
A1	20 (53%)
A2	13 (34%)
A3	5 (13%)
A4	0
8 months: (N = 142)	35 (25%)
A1	20 (57%)
A2	13 (37%)
A3	1 (3%)
A4	1 (3%)
12 months: (N = 132)	26 (20%)
A1	16 (61%)
A2	9 (35%)
A3	1 (4%)
A4	0
18 months: (N = 119)	17 (14%)
A1	12 (71%)
A2	5 (29%)
A3	0
A4	0
24 months: (N = 103)	20 (20%)
A1	14 (70%)
A2	4 (20%)
A3	2 (10%)
A4	0
FEV1 at 24 months	79 ± 23
FVC at 24 months	87 ± 23
24-month mortality	34 (19%)

Legend: The notation A1–A4 refers to the histological grading of acute cellular rejection in lung transplantation, as defined by the International Society for Heart and Lung Transplantation (ISHLT). This classification is part of the revised working formulation for the standardization of nomenclature in the diagnosis of lung rejection [21].

**Table 2:** Clinical characteristics of HHV-6 positive patients in at least one BAL surveillance, in different time points.

Variable	1 Month		4 Months		8 Months		12 Months		18 Months		24 Months	
	N = 146	*p*-Value *	N = 159	*p*-Value *	N = 147	*p*-Value *	N = 140	*p*-Value *	N = 121	*p*-Value *	N = 109	*p*-Value *
24-month mortality	20 (41)	0.449	20 (43)	0.863	21 (39)	0.880	19 (38)	0.408	11 (29)	0.997	7 (22)	0.981
Post-tx PTLD	-	-	2 (4)	0.028	2 (4)	0.620	2 (4)	0.056	-	-	-	-
CMV +	20 (41)	0.420	36 (78)	0.006	39 (72)	0.179	34 (68)	0.188	30 (79)	0.075	24 (75)	0.061
EBV +	27 (55)	<0.001	16 (35)	0.010	24 (44)	0.009	23 (46)	0.003	18 (47)	0.297	17 (53)	0.010
HSV 1	13 (27)	0.070	7 (15)	0.180	2 (4)	0.351	4 (8)	0.769	1 (3)	0.425	7 (22)	0.003
Bacteria pos.	20 (42)	0.070	15 (32)	0.724	8 (15)	0.155	13 (26)	0.724	9 (24)	0.848	8 (25)	0.767
*A.xylosoxidans*	6 (13)	0.010	-	-	-	-	2 (4)	0.515	-	-	-	-
Fungi	7 (15)	0.337	2 (4)	0.242	3 (6)	0.499	3 (6)	0.718	2 (5)	0.183	1 (3)	0.122
Bacterial and fungi co-infection	5 (11)	0.008	0	0.106	0	0.445	1 (2)	0.660	-	-	1 (3)	0.122
Acute rejection at TBB	16 (37)	0.202	11 (24)	0.993	11 (22)	0.795	4 (9)	0.073	4 (11)	0.705	24 (75)	0.061
FEV 1	70 ± 19	0.861	72 (24)	0.306	80 (20)	0.281	77 (23)	0.658	82 (19)	0.370	81 (23)	0.589
FVC	69 ± 20	0.852	77 (26)	0.151	87 (24)	0.307	84 (23)	0.698	87 (18)	0.983	88 (23)	0.744

* *p*-value for HHV6 positive vs. negative patients. Legend: Post-tx PTLD: Post-transplant lymphoproliferative disorder, CMV: cytomegalovirus, EBV: Epstein–Barr virus, HSV 1: herpes simplex virus type 1, TBB: transbronchial biopsy, FEV 1: forced expiratory volume in one second, and FVC: forced vital capacity.

**Table 3:** Spirometry data FEV1 and FVC percentage of predicted (median ± SD).

Variable	1 Month	4 Months	8 Months	12 Months	18 Months	24 Months
FEV1 HHV6 neg.	73 ± 14	84 ± 17	84 ± 22	84 ± 23	86 ± 22	86 ± 25
FEV 1 HHV6 pos.	71 ± 18	79 ± 19	82 ± 18	83 ± 20	82 ± 20	81 ± 21
FEV1 *p*-value	0.683	0.244	0.692	0.836	0.508	0.360
FVC HHV6 neg.	73 ± 13	92 ± 14	98 ± 16	98 ± 20	100 ± 21	100 ± 21
FVC HHV6 pos.	73 ± 17	82 ± 19	87 ± 18	89 ± 18	90 ± 19	87 ± 19
FVC *p*-value	0.507	0.038	0.033	0.094	0.064	0.038

## Data Availability

Data is available upon request.
